# Metabolite-enabled mutualistic interaction between *Shewanella oneidensis* and *Escherichia coli* in a co-culture using an electrode as electron acceptor

**DOI:** 10.1038/srep11222

**Published:** 2015-06-10

**Authors:** Victor Bochuan Wang, Krishnakumar Sivakumar, Liang Yang, Qichun Zhang, Staffan Kjelleberg, Say Chye Joachim Loo, Bin Cao

**Affiliations:** 1 School of Materials Science and Engineering, Nanyang Technological University, Singapore 639798, Singapore; 2 School of Civil and Environmental Engineering, Nanyang Technological University, Singapore 637511, Singapore; 3 School of Biological Sciences, Nanyang Technological University, Singapore 637551, Singapore; 4 Singapore Centre on Environmental Life Sciences Engineering (SCELSE), Nanyang Technological University, Singapore 637551, Singapore; 5 Singapore Centre on Environmental Life Sciences Engineering (SCELSE), Interdisciplinary Graduate School, Nanyang Technological University, Singapore 639798, Singapore; 6 School of Biotechnology and Biomolecular Sciences and Centre for Marine Bio-Innovation, The University of New South Wales, Sydney NSW 2052, Australia

## Abstract

Mutualistic interactions in planktonic microbial communities have been extensively studied. However, our understanding on mutualistic communities consisting of co-existing planktonic cells and biofilms is limited. Here, we report a planktonic cells-biofilm mutualistic system established by the fermentative bacterium *Escherichia coli* and the dissimilatory metal-reducing bacterium *Shewanella oneidensis* in a bioelectrochemical device, where planktonic cells in the anode media interact with the biofilms on the electrode. Our results show that the transfer of formate is the key mechanism in this mutualistic system. More importantly, we demonstrate that the relative distribution of *E. coli* and *S. oneidensis* in the liquid media and biofilm is likely driven by their metabolic functions towards an optimum communal metabolism in the bioelectrochemical device. RNA sequencing-based transcriptomic analyses of the interacting organisms in the mutualistic system potentially reveal differential expression of genes involved in extracellular electron transfer pathways in both species in the planktonic cultures and biofilms.

Mutualistic interaction between microorganisms involves closely coupled cell-cell associated interactions in microbial communities and plays critical roles in biogeochemical cycles, waste decomposition and biofuel production^1^. In mutualism, interactions between two microbial species occur to bring about benefits to each species in the relationship^2^. Recently, increased research efforts have been dedicated to the development of models to describe mutualistic interactions in bacterial communities^3^,^4^. Diverse interactions have been observed to drive mutualistic systems. These include a resource-service system, which is a common mutualistic model where one species produces a chemical compound which is consumed by another species for mutual benefits^5^. The collective output through mutualistic interactions is typically greater than that of each of the monoculture systems. For example, a defined binary culture of the cellulolytic fermenter, *Clostridium cellulolyticum,* and the electrochemically active *Geobacter sulfurreducens* respires on an extracellular electrode to convert a specific cellulosic biomass to useful energy^6^. In another study, the physiological change and biofilm structure of *Shewanella oneidensis* were similar in pure- and co-culture bioelectrochemical systems, when coupled to the homolactic fermenter *Lactococcus lactis*, pointing towards a purely food-web relationship at the substrate level^7^. Most of the studies to date have focused on mutualistic interactions in planktonic cultures and are still lacking in knowledge of the identity of the metabolite(s) of exchange for enhanced extracellular electron transfer and fundamental interactions of the involved species in the established community. In most natural and engineered settings, planktonic cells often coexist with biofilms, which are structured, surface-associated microbial communities that are prevalent in various natural, engineered and medical habitats. Hence, there is a need to elucidate mutualistic interactions in microbial communities consisting of planktonic cells interacting with biofilms. To this end, a planktonic cells-biofilm mutualistic system, established by a fermentative bacterium and a metal-reducing bacterium in a bioelectrochemical device, is explored in this contribution.

*Escherichia coli* was chosen as a representative fermentative microorganism as it is easy to culture with simple nutritional requirements and its genome sequence is relatively well annotated^8^, while *S. oneidensis*, a well-studied metal-reducing bacterium capable of reducing a wide range of metal ions, was used as a model anaerobically respiring bacteria. In addition, *S. oneidensis* produces soluble electron mediators such as flavins, which can facilitate electron transfer between outer membrane cytochromes and solid electron acceptors.

Here, using *E. coli* and *S. oneidensis* as model organisms, we report the establishment of biofilm-mediated mutualistic interactions between fermentative anaerobic bacteria and dissimilatory metal-reducing bacteria in bioelectrochemical devices, such as microbial fuel cells (MFCs). We further demonstrate that the assembly of the mutualistic structure is driven by specific microbial functions of the interacting microorganisms. RNA sequencing revealed up-regulation of certain extracellular electron transfer genes in the interacting organisms from the mutualistic system.

## Results and Discussion

### Mutualistic interaction is established in MFCs

**Figure 1:**
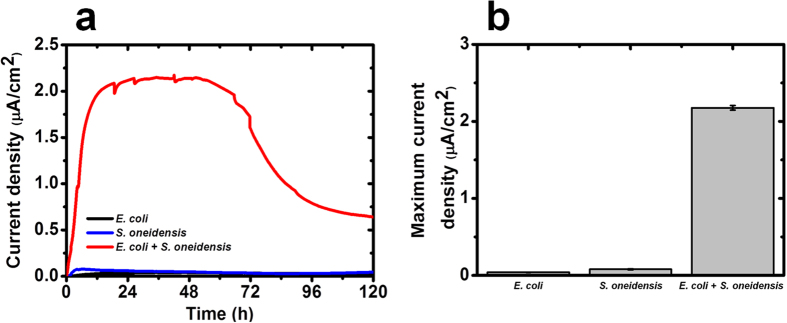
Electrical output of MFCs inoculated with mono- and co-cultures of *E. coli* and *S. oneidensis* microbial strains. (**a**) Current density generated as a function of time. (**b**) Maximum current density (average ± standard deviation). Data represent the average of triplicates.

To test the mutualistic co-culture relationship between wild type *E. coli* and *S. oneidensis* strains, MFCs were employed as a platform to determine the extent of extracellular electron transfer in terms of electrical output in bioelectrochemical devices, with both species inoculated into the anode chamber. MFCs were operated for each experimental parameter and the average generated current densities by each of the combination of wild type or mutant strains of *S. oneidensis* were recorded (Fig. 1a). The co-culture MFCs produced a significantly higher average maximum current density (~2.0 μA/cm^2^), whereas mono-culture MFCs produced negligible current densities (Fig. 1b). These findings suggest that there were mutualistic interactions between *E. coli* and *S. oneidensis*. Further, the experiment revealed a rapid increase (~0.17 μA/cm^2^/h) in the average current density generated by the co-culture, suggesting an immediate establishment of the mutualistic interaction.

### Formate is identified as the main metabolite of exchange in the mutualistic co-culture system

**Figure 2:**
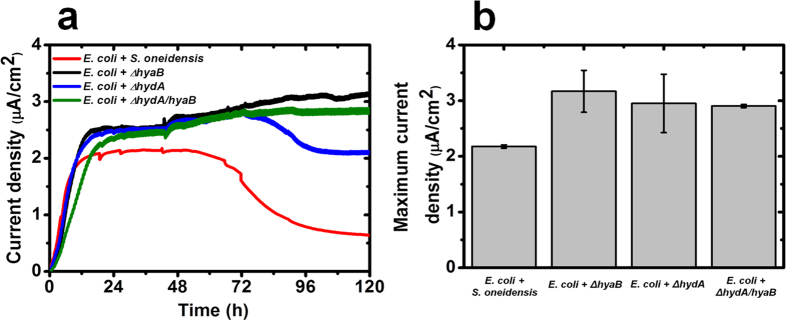
Electrical output of MFCs inoculated with co-cultures of *E. coli*, *S. oneidensis* and mutant hydrogenases microbial strains. (**a**) Current density generated as a function of time. (**b**) Maximum current density (average ± standard deviation). Data represent the average of triplicates.

Hydrogen and formate are common metabolites of exchange in mutualistic systems and are produced from glucose fermentation by *E. coli*^9^. It has been shown that hydrogen transfer can mediate electron flow in mutualistic systems efficiently through hydrogen utilization catalyzed by hydrogenases^1^. To investigate the role of hydrogen transfer in our MFC-based mutualistic system, *S. oneidensis* mutant strains lacking hydrogenases were used to interact with *E. coli*^10^. MFCs containing co-cultures of *E. coli* and *S. oneidensis* hydrogenase mutants generated average maximum current densities of ~3.0 μA/cm^2^ (Fig. 2a,b), which is ~33% than that of the wild type co-cultures, suggesting that hydrogen is not a key metabolite of exchange in this mutualistic system. Based on additional experiments, the specific growth rate of wild type *S. oneidensis* and hydrogenase mutants are similar. However, towards the end of the stationary phase (~24 h), the hydrogenase mutants exhibited a higher biomass than the wild type (OD_600−_ wild type: 0.327 ± 0.0050; *ΔhydA*: 0.369 ± 0.0100; *ΔhyaB*: 0.505 ± 0.0148; *ΔhydA/hyaB*: 0.438 ± 0.0018). The higher current density exhibited by the hydrogenase mutants could be attributed to the higher cell yield.

**Figure 3:**
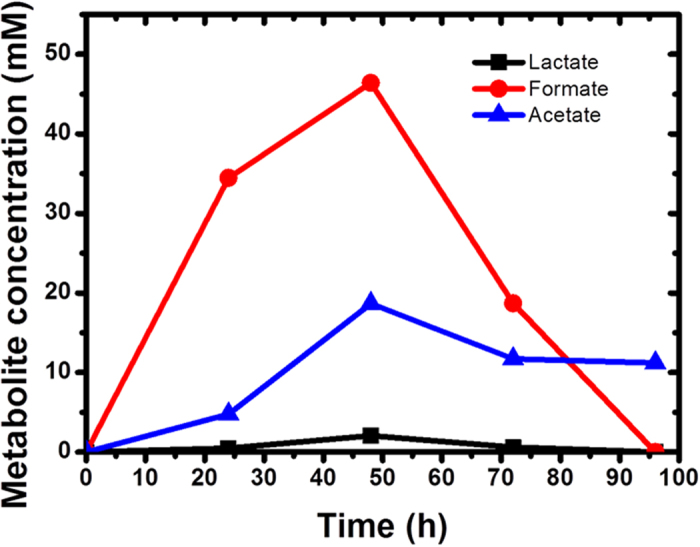
Concentration of lactate, formate and acetate in MFC anode chamber containing the mutualistic system. Experiments were performed in different batches but all results are in agreement.

To further elucidate the metabolite of exchange, HPLC was used to quantify the key metabolites in the mutualistic system. Formate, acetate and lactate were identified as the three main metabolites (Fig. 3). The concentration of lactate was significantly low (<1.0 mM) throughout the MFC operation. Acetate concentration remained relatively constant (~10.0 mM) as it cannot be utilized by *S. oneidensis*. In contrast, the concentration of formate increased rapidly to ~45.0 mM over 48 h and decreased thereafter to ~0 mM within 100 h. This observation agrees well with the electrical output of co-culture MFCs, which started to decrease after ~48 h (Fig. 1a). Hydrogen quantification in the headspace revealed no significant change in co-culture systems with wild type and mutant hydrogenase species after 72 h (Figure S1). Taken together, our results suggest that formate is the main metabolite of exchange in this MFC-based mutualistic system.

### Increase in extracellular flavins enhances the current generation in the mutualistic system

Riboflavins and flavin mononucleotides (FMN) secreted by *S. oneidensis* could be significant factors contributing to high current density in the mutualistic system as these redox molecules are capable of functioning as electron shuttles^11^,^12^,^13^. Further, previous research has revealed that flavins can act as mediators in *S. oneidensis* without direct contact^11^,^14^,^15^. To elucidate whether flavins are critical in generating high current density in the mutualistic system, the concentration of extracellular flavins were quantified in monoculture and mutualistic systems. The highest flavins concentrations were observed in mutualistic co-culture systems after ~24 h, reaching up to ~25 ± 2.44 nM, whereas monoculture systems produced significantly lesser flavins (*E. coli*: 8.0 ± 0.6 nM and *S. oneidensis*: 8.2 ± 0.3 nM) after ~24 h. The presence of low concentrations of extracellular flavins in *E. coli* monocultures may in part be linked to a certain degree of cell lysis, leading to release of flavins. The enhanced current generation observed in the mutualistic system may thus be attributed to increased extracellular flavins secreted by *S. oneidensis*, which is consistent with previous reports^11^,^16^,^17^. It has also been observed that the addition of flavins boost the current production in *E. coli* monoculture systems^18^. A mutualistic interaction in a MFC co-culture of fermentative bacterium and a metal-reducing bacterium has been reported, where *Pelobacter carbinolicus* fermented ethanol to hydrogen and acetate for *G. sulfurreducens* to consume, and in turn, *G. sulfurreducens* consumed the hydrogen to reduce the partial pressure in the system for *P. carbinolicus* to facilitate its fermentation process^19^. However, in the *P. carbinolicus*/*G. sulfurreducens* system, extracellular electron transfer from the co-culture to the electrode was solely achieved by *G. sulfurreducens*, while *P. carbinolicus* only provided electron donors for *G. sulfurreducens*. Our results demonstrate a novel type of mutualistic interaction between *S. oneidensis* and *E. coli*, in which flavins secreted by *S. oneidensis* facilitate flavin-mediated electron transfer from *E. coli* to the anode while *E. coli* ferments glucose to produce formate for *S. oneidensis* biofilms on the electrode to use as an electron donor.

### Mutualistic interactions determine the community structure across anode media and electrode

**Figure 4:**
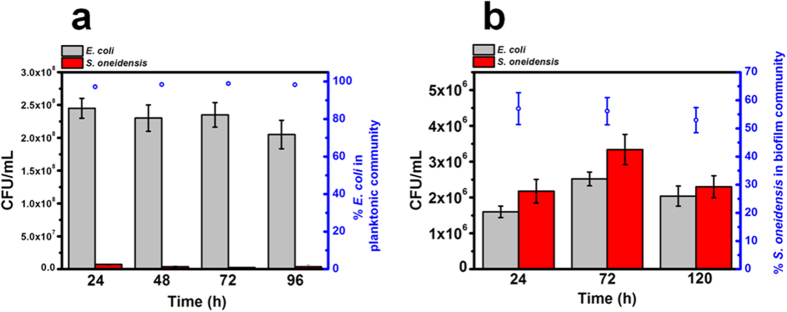
Community dynamics in the MFC-based mutualistic system. (**a**) CFU counts and relative proportion (%) of *E. coli* and *S. oneidensis* strains in anode media. (**b**) CFU counts and relative proportion (%) of *E. coli* and *S. oneidensis* strains on the anode electrode.

Community dynamics and interactions between specific bacterial species are fundamental in determining how mutualistic systems function as an entity in various ecological settings^3^,^4^,^6^. To elucidate the structure-function relationship of the mutualistic system, we quantified the community dynamics in terms of CFU and biovolume of samples from the anode media and the electrodes. Although both *E. coli* and *S. oneidensis* are classically shown to be good biofilm forming organisms^20^,^21^,^22^,^23^, we found that *E. coli* represented the majority of the cells (~98%) (Fig. 4a) in the planktonic fraction of the MFC-based mutualistic community, whereas *S. oneidensis* dominated the biofilms on the electrode (biovolume fraction ~ 60%) (Fig. 4b). It is noteworthy to mention that the electrode serves as the sole electron acceptor for *S. oneidensis* respiration while the only carbon source (glucose) for *E. coli* fermentation is in the aqueous media. This observation implies that the distribution of each species in the mutualistic community could be driven by metabolic functions towards an optimum communal metabolism (Fig. 4a,b). It has been reported that the combination of fermentation and anaerobic respiration across different species is more thermodynamically favorable than a sole fermentative process by a single bacterial species, based on the energy yield per electron transferred in the respective reactions^24^,^25^. Hence, the establishment of a dual species biofilm community of *E. coli* and *S. oneidensis* on the electrode may have an advantage over mono-species biofilms formed by *E. coli*. Over the course of the MFCs operation, *S. oneidensis* was preferentially localized on the anode electrode (Fig. 4b), suggesting that it has developed an ecological niche at the locale of the electrode, probably due to its capability to utilize formate from glucose fermentation by *E. coli* coupled to the respiration of the anode electrode^13^,^26^,^27^.

To understand how communal metabolism can be harnessed by the mutualistic system studied here, a material balance was performed on the glucose fermentation by *E. coli* planktonic cells. Based on the dry *E. coli* weight (derived from the CFU count in Fig. 4a) and biomass yield of *E. coli* in glucose fermentation^28^, it was revealed that ~60% of glucose is consumed by *E. coli* planktonic cells, leading to the release of fermentation products. The number of electrons transferred from *S. oneidensis* to the electrode is equivalent to the number of electrons released by the oxidation of formate and lactate by *S. oneidensis*. Based on the generated concentrations of formate and lactate (Fig. 3), it was observed that the number of electrons derived from *S. oneidensis* varied from 4.36 × 10^17^ (24 h), 6.05 × 10^17^ (48 h) and 2.41 × 10^17^ (72 h), which accounted for ~3–4% of the total electrons derived from the co-culture system (Fig. 2a). The calculations to elucidate the contribution of electrons from each species are based on the supporting equations for glycolysis pathway of fermentation, which are provided in Supplementary Information. This suggests that the majority of electrons (~96–97%) are derived from *E. coli,* which utilizes flavins as electron shuttles. This points towards flavins-mediated electron transfer as the main extracellular electron transfer mechanism in the co-culture system. These results provide a novel perspective for mutualistic interactions. Although *E. coli* is considered an inferior electrogenic bacterium, the presence of *S. oneidensis* improves the electrogenic properties of *E. coli* through the release of flavins. This in turn releases simple metabolites for *S. oneidensis* to undergo anaerobic respiration. Hence, the mutualistic community which has been enriched with species of diverse metabolic functions is able to drive itself towards an optimum communal metabolism.

### Preferential localization of *S. oneidensis* cells on electrode favours mutualistic interaction

To further understand the preferential localization of both species, the RNA-sequencing based transcriptomic approach was employed to study the effect of the mutualistic relationship in *S. oneidensis* biofilms formed on the electrodes. Gene expression profiles of extracellular electron transfer related genes (MTR pathway and flavins biosynthesis) are specifically targeted. In this co-culture system, such genes in the context of *S. oneidensis* biofilm are revealed to be up-regulated (Table 1). In particular, expression of genes involved in the MTR pathway (*cymA, mtrA, mtrC, omcA* and *mtrB*) and flavin biosynthesis (*fccA, frdA, SO 3468* and *ribE*) are notably increased. These observations suggest a likely advantage for *S. oneidensis* biofilms to preferentially form on the electrode surface as it beneficially contributes to the communal interactions between both species in the area of charge transfer. This corroborates the CFU and biovolume data presented previously (Fig. 4b), which illustrates a stronger *S. oneidensis* biofilm presence in the community. In addition, gene expression of formate dehydrogenase genes (*fdhA*, *fdhB*, *fdhT* and *fdhX*) are also significantly increased, which strongly suggests that formate is the main metabolite of exchange (Fig. 3).

### Transcriptomic responses of *E. coli* to removal of formate by *S. oneidensis*

As *S. oneidensis* consumes formate for its own metabolism, a ‘sink’ is formed in the co-culture system. Transcriptomic data reveals that genes involved in metabolism in *E. coli* are up-regulated (Table 2). This suggests a mutualistic relationship, where *S. oneidensis* takes up formate for respiration, which releases electrons, whereas *E. coli* ferments glucose to produce formate as a form of metabolism. In addition, *YdiQ*, which is a flavoprotein, is also significantly up-regulated in *E. coli*, which suggests that, other than formate removal by *S. oneidensis*, secreted flavins from *S. oneidensis* can potentially induce increased *E. coli* metabolism.

### Proposed mutualistic interaction model between *E. coli* and *S. oneidensis*

**Figure 5:**
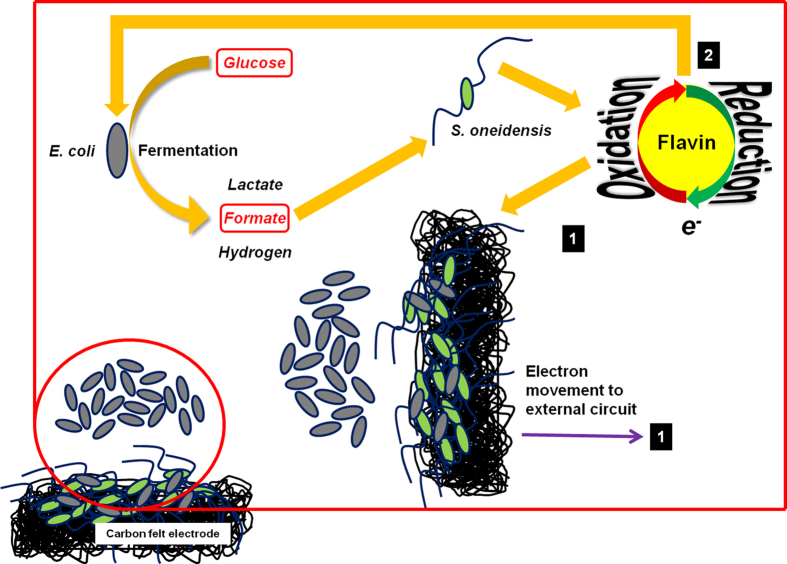
Model of the mutualistic relationship between *E. coli* and *S. oneidensis*. Glucose is fermented by *E. coli* to yield metabolites such as formate, which is taken up by *S. oneidensis* as electron donor. Flavin mediator molecules are secreted to facilitate electron movement to the external charge collecting electrode to derive energy in MFCs. Flavins can also be taken up by *E. coli* to facilitate its respiration on electrodes as *E. coli* does not secrete flavins.

Based on the results obtained from this study, we propose a mutualistic interaction model between fermentative *E. coli* and dissimilatory metal reducing *S. oneidensis* (Fig. 5). *S. oneidensis* is unable to metabolize glucose in the anode media, while *E. coli* ferments glucose to produce metabolites including formate, which is selectively utilized by *S. oneidensis* as an electron donor in MFCs, where the anode electrode serves as the terminal electron acceptor. *S. oneidensis* produces flavins that can facilitate the electrode reduction. In addition, these electron mediators can also enhance the metabolic activity of *E. coli* in the mutualistic community^13^,^29^,^30^. It is estimated that ~60% of glucose was consumed by the planktonic *E. coli* cells. ~4% of the electrons derive from the *S. oneidensis* biofilms on the electrode and the remaining ~96% are generated by *E. coli* which utilizes flavins secreted by *S. oneidensis* to facilitate extracellular electron transfer.

In summary, we have established a mutualistic system between fermentative *E. coli* and the dissimilatory metal reducing *S. oneidensis* in MFCs with interacting planktonic culture in the anode media and biofilms on the electrodes. We showed that the transfer of formate is the key mechanism in this mutualistic system. Further, we demonstrated that the structural assembly of this mutualistic community is driven by metabolic functions towards an optimum communal metabolism. This study reports a function-driven structural assembly of mutualistic communities consisting of planktonic cells interacting with biofilms.

## Methods

### Bacterial strains and growth conditions

Bacterial strains used in this study are listed in Table 3. *S. oneidensis* MR-1 and mutants were kindly provided by Dr. Liang Shi and Dr. Jim Fredrickson from Pacific Northwestern National Laboratory. *E. coli* K-12 (ATCC #10798) was purchased from the American Type Culture Collection (Manassas, VA). Stock cultures were stored in lysogeny broth (LB) medium with 25% glycerol at –80 °C. Monocultures of *E. coli* or *S. oneidensis* for inoculation of MFCs were prepared aerobically in LB medium at 37 °C and 30 °C, respectively.

### Growth media for co-cultures in MFCs

The media in the anode chamber of the MFCs was a 1:1 (v/v) mixture of M9 and M1 salt solution, supplemented with 20 mM glucose as the sole carbon source. The M9 salt solution consisted of 64 g/L of sodium monohydrogen phosphate heptahydrate, 15 g/L of monopotassium phosphate, 2.5 g/L of sodium chloride and 5 g/L of ammonium chloride. The M1 salt solution consisted of 7.2 g/L of HEPES, 0.3 g/L of sodium hydroxide, 1.5 g/L of ammonium chloride, 0.1 g/L of potassium chloride, 0.52 g/L of monosodium phosphate, and trace amounts (1% v/v) of mineral stock solution, vitamin stock solution and amino acids stock solution^31^. Before use, 2 mL of 1 M magnesium sulphate and 100 μL of 1 M calcium chloride were added to the media to final concentrations of 2 mM and 100 μM respectively.

### MFC set-up

All materials were used as received, unless otherwise stated. Glass tubes (17 mm O. D. × 1.8 mm wall thickness) forming the anode and cathode chambers of the MFCs, carbon felt (3.18 mm thickness) and stainless steel pinch clamps (#28) were purchased from VWR Singapore Pte. Ltd. (Singapore). Titanium wire (0.25 mm diameter), Nafion® N117 proton exchange membrane (PEM) and serrated silicone septa (18 mm O. D.) were purchased from Sigma-Aldrich, Singapore (Singapore). Nylon screws and nuts were purchased from Small Parts, Inc. (United States of America).

**Figure 6:**
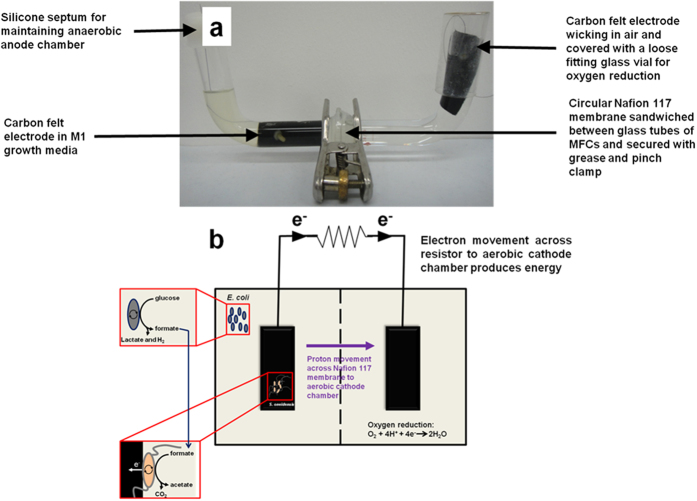
Illustration of MFC setup used to investigate mutualistic relations. (**a**) Photograph of MFC setup. (**b**) Graphic of mutualistic interactions between *E. coli* and *S. oneidensis* in MFC setup.

Dual chamber U-tube MFCs were constructed as reported previously^32^,^33^,^34^. An illustration of an actual MFC containing the mutualistic relations is shown (Fig. 6). Two 90° 28/15 ball-to-plain-end and socket-to-plain-end glass tubes were separated from each other by a piece of Nafion® N117 PEM. The joints of the glass tubes were greased and sealed against a circular piece of PEM (diameter of 2 cm). The whole assembly was held in place and tightened with a stainless steel pinch clamp. Carbon felt electrodes were cut to 2 cm × 5 cm dimensions (width × length) and fastened to the titanium wire with the nylon screw and nut. The assembled electrodes were then seated inside the glass tubes. Prior to MFC operation, the devices were filled with ultrapure water and autoclaved to sterilize the devices and internal components. After sterilization and decanting off the ultrapure water, the anode and cathode chambers were each filled with the growth medium for co-cultures. 1 mL overnight culture (OD_600_ ~ 1.0) for each bacterial strain was then inoculated into the anode chamber only. The final total volume of solution in each of the anode and cathode chambers was 20 mL. The anode chamber was sealed with a serrated silicone septum through which the titanium wire was threaded, while the cathode chamber was loosely capped with an inverted glass scintillation vial to provide an aerobic environment. The cathode electrodes were only partly submerged in the catholyte to allow for an ‘air-wicking’ aerobic configuration. The electrodes were then connected to a 1 kΩ resistor and voltage measurements across the resistors were recorded at a rate of 1 point per 5 minutes using an eDAQ e-corder® data acquisition system (Bronjo Medi, Singapore), equipped with Chart® software. Data collection started immediately after inoculation of the devices. MFCs were operated in triplicates and kept inside an incubator set to 33 °C for up to 5 days.

### Cell harvesting for RNA extraction

Representative electrodes from specific MFCs were transferred to 15 mL centrifuge tubes and mixed immediately with 2 mL of RNAprotect® Bacteria Reagent (Qiagen) to fully immerse the electrode. The mixtures were then put through vigorous vortex for 2 minutes to dislodge the biofilm cells. After 5 minutes of incubation at room temperature, the supernatant containing the cells were removed from the electrode and placed in micro-centrifuge tubes. The samples were centrifuged at 7000 g for 12 minutes at 4 °C and the supernatant was removed, leaving only the cell pellet. The cell pellets were stored at –80 °C until further experiments.

### RNA extraction

The bacterial cell pellets were thawed in ice and treated with lysozyme in TE buffer. Total RNA was extracted with RNeasy Mini Purification kit (Qiagen). On-column DNase digestion with the RNase-free DNase Set (Qiagen) was carried out to facilitate the removal of DNA. The concentration of RNA and presence of DNA contamination was assessed using a Qubit® 2.0 Fluorometer (Invitrogen). The integrity of RNA was assessed with the Agilent 2200 TapeStation System (Agilent Technologies).

### RNA Sequencing

The quality of the RNA samples was confirmed with the Quant-iT™ RiboGreen® RNA Assay Kit (Invitrogen) and Quant-iT™ PicoGreen® dsDNA Assay Kit (Invitrogen) on a Bioanalyzer RNA 6000 Nano Chip (Agilent). Subsequently, next-generation sequencing library was prepared by adopting the TruSeq RNA Sample Preparation v2 protocol (Illumina) with modifications. mRNA purification was excluded and 200 ng of total RNA was added to the elute-fragment-prime step instead. PCR was amplified stepwise, which enriches selectively for library fragments with adapters ligated on both ends. These steps were executed according to the manufacturer’s protocol but the amplification cycles were minimized to 12 steps. Each library was specifically tagged with barcodes from Illumina’s TruSeq LT RNA to facilitate library pooling for sequencing. Library quantization was produced using Invitrogen’s Picogreen assay. The average library size was confirmed by referencing the libraries on a Bioanalyzer DNA 1000 chip (Agilent). The library concentration was adjusted to 2 nM and the concentration was determined by qPCR on a ViiA-7 real-time thermocycler (Applied Biosystems), employing qPCR primers in Illumina’s qPCR protocol and Illumina’s PhiX control library as a standard. All the libraries were then combined equally and sequenced in two lanes of an Illumina HiSeq2500 rapid run at a final concentration of 7.2 pM and a read-length of 101 bp paired-end.

### Computational Analysis

The Illumina RNA sequencing raw data were deposited in the NCBI Sequence Read Archive database (http://www.ncbi.nlm.nih.gov/sra) with an accession number SRP056131 (Bio-Project number: PRJNA277919). The combined sequence reads were analyzed in RNA-Seq and expression analysis application of CLC genomics Workbench 6.0 (CLC Bio, Aarhus, Denmark). The *E. coli* and *S. oneidensis* genome were utilized as reference genomes for the analysis. These conditions were used to filter the unique sequence reads: minimum length fraction of 0.9, minimum similarity fraction of 0.8, and maximum number of 2 mismatches. Data were adjusted by calculating the reads per kilobase per million mapped reads (RPKM) for each gene^35^. Results were annotated using respective databases. Statistical analyses ANOVA and t-test (p ≤ 0.05) were employed and the fold change ratio (R) of each gene expression was calculated as the ratio of RPKM in treated sample with respect to the control. The entire transcriptomics data has been included in Supplementary Information (Tables S1 and S2).

### CLSM imaging to estimate biovolume of biofilms on electrode

Carl Zeiss Confocal Laser Scanning Microscopy (CLSM) model LSM 780 was used to acquire images of the carbon felt electrodes, which were colonized by GFP-tagged *S. oneidensis* and *E. coli*. The biofilms on electrodes were stained with 4’,6-diamidino-2-phenylindole (DAPI). GFP and DAPI image channels were illuminated with 488 nm and 405 nm laser excitation respectively. GFP and DAPI images were acquired simultaneously on two detection channels with the GFP channel for *S. oneidensis* cells while the DAPI channel was for both *E. coli* and *S. oneidensis* cells. Image stacks were acquired using 20 × /0.4 N.A. objective lens. The images were then re-constructed using the software package Zen 2011 (Carl Zeiss, Singapore). IMARIS (Bitplane AG, Zurich, CH) was used to estimate biovolume based on the reconstructed three-dimensional images.

### Determination of the community structure

The community structure of the biofilms on the electrode and the planktonic culture in the anode was determined. For biofilms on the electrode, a small representative section of the electrode, periodically retrieved from the anode chamber of the co-culture MFCs, was rinsed with sterile M1-M9 salt solution (1:1 v/v) and scrapped with a sterile surgical blade into 2 mL of M1-M9 solution. Samples from both re-suspended biofilms and the planktonic culture were vigorously vortexed, followed by serial dilution for colony forming unit (CFU) counts using a drop-plate method described elsewhere^36^. Fluorescent colonies observed in the dark by using a fluorescent torch (MeCan Imaging Inc., Japan) were attributed to the GFP-tagged *S. oneidensis* cells. *E. coli* colonies were counted by subtracting fluorescent colonies from the total amount of colonies.

### Glucose utilization by planktonic E. coli cells

A glucose fermentation batch study was conducted to establish the correlation between *E. coli* CFU/mL versus dry weight. The media used for this study was the growth medium for co-cultures in MFCs, as described above. Samples were withdrawn at regular intervals for CFU counts, followed by centrifugation and filtration to prepare cell-free supernatants for glucose and flavin quantification. The *E. coli* biomass yield in glucose fermentation (Y_glucose_) was taken to be 0.524 for the growth phase (24 h)^28^,^37^. The glucose consumption by the *E. coli* planktonic cells in MFCs was estimated based on the *E. coli* planktonic cell density in terms of dry weight and the biomass yield of *E. coli* in glucose fermentation^28^. The number of Coulombs of electrons collected from the respective MFCs was calculated by integrating the current (A) versus time (s) graphs.

### Metabolite analysis

The extracellular metabolites (e.g., lactate, formate and acetate) were quantified over the operational time of the MFCs by using a high performance liquid chromatography (HPLC) system, equipped with a HPX-87 H (Bio-Rad) ion exchange column (300 mm × 7.8 mm) and UV detector with 8 mM H_2_SO_4_ as the mobile phase at a flow rate of 0.6 mL/min^38^,^39^. The glucose concentration in the system was observed using the same column and refractive index detector with 0.008 M H_2_SO_4_ as the mobile phase.

### Flavin quantification

For fluorescence measurements, 200 μL of the cell free supernatant was transferred to a clear 96 well plate and read at 440 nm excitation and 525 nm emission^40^. The background fluorescence was corrected by using the selective minimal media as the blank.

## Additional Information

**How to cite this article**: Wang, V. B. *et al.* Metabolite-enabled mutualistic interaction between Shewanella oneidensis and Escherichia coli in a co-culture using an electrode as electron acceptor. *Sci. Rep.* **5**, 11222; doi: 10.1038/srep11222 (2015).

## Supplementary Material

Supplementary Information

## Figures and Tables

**Table 1:** **Selected gene expression changes in** ***S. oneidensis*** **– biofilm on electrode versus planktonic culture in anode chamber.**

**Extracellular electron transfer pathway – MTR pathway**		
**Gene**	**Function**	**log_2_(fold change)**
*cymA*	Membrane anchored tetraheme cytochrome c	0.73
*mtrA*	Extracellular iron oxide respiratory system periplasmic decaheme cytochrome c component	2.65
*mtrC*	Extracellular iron oxide respiratory system surface decaheme cytochrome c component	0.75
*omcA*	Extracellular iron oxide respiratory system surface decaheme cytochrome c component	1.39
*mtrB*	Extracellular iron oxide respiratory system outer membrane component	2.72
**Extracellular electron transfer pathway – Flavin biosynthesis**		
**Gene**	**Function**	**log**_**2**_**(fold change)**
*fccA*	Periplasmic fumarate reductase	0.78
*frdA*	Quinol:fumarate reductase FAD-binding subunit	0.97
*SO 3468*	Riboflavin synthase alpha subunit RibC-like protein	4.11
*ribE*	Riboflavin synthase beta subunit RibE	1.26
**Formate dehydrogenase genes**		
**Gene**	**Function**	**log**_**2**_**(fold change)**
*fdhA*	Formate dehydrogenase	3.03
*fdhB*	Formate dehydrogenase	5.81
*fdhT*	Formate dehydrogenase	1.23
*fdhX*	Formate dehydrogenase	6.75

Note: Samples were collected from three biological replicates.

p value less than 0.05 were considered significant.

**Table 2:** **Selected gene expression changes in** ***E. coli*** **induced by** ***S. oneidensis*** **for energy metabolism.**

**Planktonic culture in anode chamber**		
**Gene**	**Function**	**log_2_(fold change)**
*nadE*	Metabolism of cofactors and vitamins	1.75
*rhmA*	Carbohydrate metabolism	1.21
**Biofilms on electrode in anode chamber**		
**Gene**	**Function**	**log**_**2**_**(fold change)**
*acpT*	Metabolism of cofactors and vitamins	1.05
*aes*	Xenobiotics biodegradation and metabolism	2.48
*ahpF*	Metabolism of NADH or NADPH	3.95
*argB*	Metabolism of amino acids	2.21
*entC*	Metabolism of cofactors and vitamins and biosynthesis of ubiquinones	1.66
*folC*	Metabolism of cofactors and vitamins and biosynthesis of folate	1.06
*gcvH*	Carbohydrate metabolism	2.95
*gcvT*	Metabolism of amino acids, cofactors and vitamins	2.20
*gldA*	Lipids metabolism	1.85
*glyA*	Metabolism of carbohydrate and biosynthesis of amino acids	1.05
*hemH*	Metabolism of cofactors and vitamins	2.54
*katG*	Metabolism of amino acids	3.97
*pfkA*	Metabolism of carbohydrate and biosynthesis of amino acids	1.08
*sufS*	Metabolism of amino acids, cofactors and vitamins	1.98
*uxuA*	Carbohydrate metabolism	1.93
*ydiQ*	Electron transfer flavoprotein	2.01

Note: Samples were collected from three biological replicates.

p value less than 0.05 were considered significant.

**Table 3:** **Bacterial strains used in this study.**

**Strains**	**Description**	**Reference**
*E. coli* K-12	Wild type fermentative strain	8
*S. oneidensis* MR-1	Manganese reducing strain (MR-1)	41,42
Δ*hydA*	*hydA* (SO3920) deletion derivative of MR-1	10
Δ*hyaB*	*hyaB* (SO2098) deletion derivative of MR-1	10
Δ*hydA/hyaB*	*hydA*-*hyaB* deletion derivative of MR-1	10
