# Supplementary material for: Metabolite-enabled mutualistic interaction between Shewanella oneidensis and Escherichia coli in a co-culture using an electrode as electron acceptor
Source: Sci Rep. 2015 Jun 10;5:11222. doi: 10.1038/srep11222 (PMC4462164; doi:10.1038/srep11222)
Supplement: Supplementary Information [file srep11222-s1.pdf]

Metabolite-enabled mutualistic interaction between *Shewanella oneidensis* and *Escherichia coli* in a co-culture using an electrode as electron acceptor

Victor Bochuan Wang[1,4^], Krishnakumar Sivakumar[5^], Liang Yang[3,4], Qichun Zhang[1], Staffan Kjelleberg[4,6], Say Chye Joachim Loo[1,4]*, Bin Cao[2,4]*

1. School of Materials Science and Engineering, Nanyang Technological University, Singapore 639798, Singapore. Email: joachimloo@ntu.edu.sg
2. School of Civil and Environmental Engineering, Nanyang Technological University, Singapore 637511, Singapore. Email: bincao@ntu.edu.sg
3. School of Biological Sciences, Nanyang Technological University, Singapore 637551, Singapore
4. Singapore Centre on Environmental Life Sciences Engineering (SCELSE), Nanyang Technological University, Singapore 637551, Singapore.
5. Singapore Centre on Environmental Life Sciences Engineering (SCELSE), Interdisciplinary Graduate School, Nanyang Technological University, Singapore 639798, Singapore.
6. School of Biotechnology and Biomolecular Sciences, The University of New South Wales, Sydney NSW 2052, Australia

*Corresponding authors

[^]Equal contribution

<u>Supplementary Information</u>

Supporting equations for electron balance calculation

Glucose → 2 [Pyruvate] → Formate or lactate

1. Glucose to pyruvate

$C_6H_{12}O_6 + 2\ ADP + 2\ P_i + 2\ NAD^+ \rightarrow 2\ CH_3COCOO^- + 2\ ATP + 2\ NADH + 2\ H_2O + 2\ H^+$

2. Pyruvate to formate

Pyruvate → Formate or lactate

3. Formate oxidation

$HCOO^- \rightarrow CO_2 + H_2O + 2e^-$

4. Lactate oxidation

$CH_3CH(OH)COO^- \rightarrow CH_3COO^- \rightarrow CO_2 + H_2O + 2e^-$

1 mole of formate or lactate oxidation release 2 moles of e^-. Hence, for the known concentration of formate, the number of electrons released can be calculated.

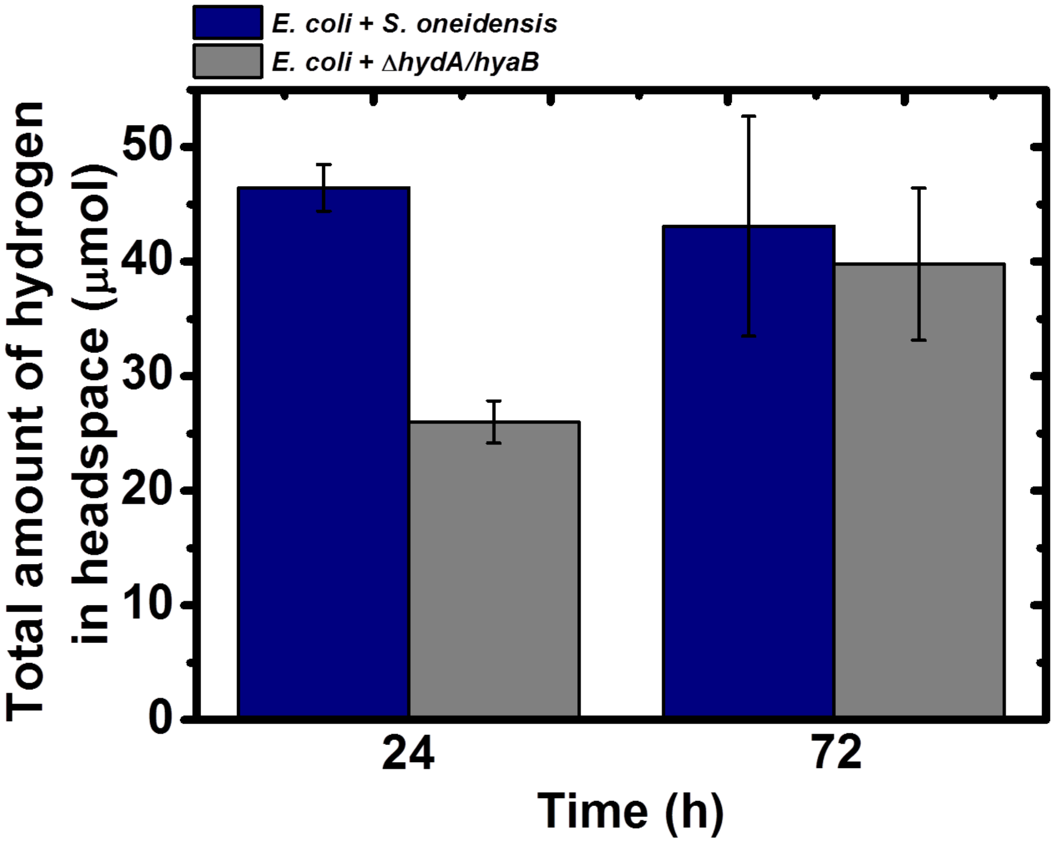

Figure S1 Quantification of hydrogen in the headspace of co-culture systems with wild type and mutant *S. oneidensis* species.

Table S1 Total gene expression changes in *E. coli* induced by *S. oneidensis*

Planktonic culture in anode chamber		
**Gene**	**Function**	**log_2 (fold change)**
abgA	P-Aminobenzoyl-Glutamate Hydrolase	-1.31
cspA	Rna Chaperone And Anti-Terminator, Cold-Inducible	-0.99
cyaY	Iron-Dependent Inhibitor Of Iron-Sulfur Cluster Formation	-1.42
cysK	Cysteine Synthase A, O-Acetylserine Sulfhydrolase A Subunit	-1.04
dnaX	Dna Polymerase Iii/Dna Elongation Factor Iii, Tau And Gamma Subunits	-1.00
flgK	Flagellar Hook-Filament Junction Protein 1	-1.40
folA	Dihydrofolate Reductase	-1.19
gnsA	Multicopy Suppressor Of Secg(Cs) And Faba6(Ts)	-1.53
metN	Dl-Methionine Transporter Subunit	-1.60
mglC	Methyl-Galactoside Transporter Subunit	-1.34
mokA	Pseudogene	-2.25
pyrE	Orotate Phosphoribosyltransferase	-2.37
narY	Nitrate Reductase 2 (Nrz), Beta Subunit	-1.08
nuoJ	NADH:Ubiquinone Oxidoreductase, Membrane Subunit J	-1.27
rimP	Ribosome Maturation Factor For 30S Subunits	-1.27
rluC	23S Rrna Pseudouridine(955,2504,2580) Synthase	-1.23
tatC	Tatabce Protein Translocation System Subunit	-1.14
tdcC	L-Threonine/L-Serine Transporter	-1.02
tdcE	Pyruvate Formate-Lyase 4/2-Ketobutyrate Formate-Lyase	-1.17
torS	Hybrid Sensory Histidine Kinase In Two-Component Regulatory System With Torr	-1.52
ubiA	P-Hydroxybenzoate Octaprenyltransferase	-1.11
waaA	3-Deoxy-D-Manno-Octulosonic-Acid Transferase (KDO Transferase)	-1.24
xisD	Pseudogene	-2.15
yafN	Antitoxin Of The Yafo-Yafn Toxin-Antitoxin System	-1.26
ybaA	Conserved Protein, DUF1428 Family	-1.68
ybeR	Predicted Protein	-1.69
ycaQ	Conserved Protein	-1.08
yceA	Predicted Rhodanese-Related Sulfurtransferase	-1.34
yciF	Predicted Rubrerythrin/Ferritin-Like Metal-Binding Protein	-1.51
ydcY	Predicted Protein	-2.88
ydfO	Qin Prophage	-1.10
yecE	Conserved Protein, UPF0759 Family	-1.03
yfbK	Conserved Protein	-1.36
yfcL	Predicted Protein	-1.99
yidB	Conserved Protein, DUF937 Family	-1.93
yigE	Predicted Protein, DUF2233 Family	-2.25
yiiG	Conserved Lipoprotein	-1.44
yjdM	Conserved Protein	-2.07
yjhX	Conserved Protein	-1.10

yjjY	Conserved Protein	-1.24
ymdA	Conserved Protein	-1.79
ymgG	Conserved Protein, UPF0757 Family	-2.35
yphG	Conserved Protein	-1.69
yqeB	Conserved Protein With Nad(P)-Binding Rossman Fold	-1.09
yrbG	Predicted Calcium/Sodium:Proton Antiporter	-1.12
nadE	Metabolism Of Cofactors And Vitamins	1.75
rhmA	Carbohydrate Metabolism	1.21
nadE	NAD Synthetase, NH3/Glutamine-Dependent	1.75
rhmA	2-Keto-3-Deoxy-L-Rhamnonate Aldolase	1.21
yaiA	Oxyr-Regulated Conserved Protein	1.02
ygbA	Predicted Protein	1.83
**Biofilms on electrode in anode chamber**		
Gene	**Function**	**log_2 (fold change)**
acpT	Holo-(Acyl Carrier Protein) Synthase 2	1.05
aes	Acetyl Esterase	2.48
ahpC	Alkyl Hydroperoxide Reductase, C22 Subunit	3.62
beeE	Copper/Silver Efflux System, Membrane Fusion Protein	1.32
argB	Acetylglutamate Kinase	2.21
cusB	Pseudogene	2.24
dps	Fe-Binding And Storage Protein	1.84
entC	Isochorismate Synthase 1	1.66
fis	Global DNA-Binding Transcriptional Dual Regulator	0.99
folC	Bifunctional Folylpolyglutamate Synthase/Dihydrofolate Synthase	1.06
gcvB	Srna Antisense Regulator Represses Oppa, Dppa, Glti And Livj Expression, Hfq-Dependent	1.82
gcvH	Glycine Cleavage Complex Lipoylprotein	2.95
gcvT	Aminomethyltransferase, Tetrahydrofolate-Dependent, Subunit (T Protein) Of Glycine Cleavage Co	2.20
gldA	Glycerol Dehydrogenase, NAD	1.85
glyA	Serine Hydroxymethyltransferase	1.05
hemH	Ferrochelatase (EC:4.99.1.1)	2.54
hflD	Predicted Lysogenization Regulator	1.06
hslU	Molecular Chaperone And Atpase Component Of Hsluv Protease	1.19
hypB	GTP Hydrolase Involved In Nickel Liganding Into Hydrogenases	1.01
insK	IS150 Transposase B	1.23
katG	Catalase-Peroxidase HPI, Heme B-Containing	3.97
livF	Leucine/Isoleucine/Valine Transporter Subunit	1.66
mntH	Manganese/Divalent Cation Transporter	2.00
motA	Proton Conductor Component Of Flagella Motor	1.85
nikA	Nickel-Binding, Heme-Binding Periplasmic Protein	1.01
nrdA	Ribonucleoside-Diphosphate Reductase 1, Alpha Subunit	1.03
nrdB	Ribonucleoside-Diphosphate Reductase 1, Beta Subunit, Ferritin-Like Protein	1.24

nrdE	Ribonucleoside-Diphosphate Reductase 2, Alpha Subunit	1.09
pfkA	6-Phosphofructokinase I	1.08
pheP	Phenylalanine Transporter	1.31
pstA	Phosphate Transporter Subunit	1.04
purD	Phosphoribosylglycinamide Synthetase Phosphoribosylamine-Glycine Ligase	1.05
purF	Amidophosphoribosyltransferase	1.55
purK	N5-Carboxyaminoimidazole Ribonucleotide Synthase	1.62
purL	Phosphoribosylformyl-Glycineamide Synthetase	1.49
sodA	Superoxide Dismutase, Mn	1.20
sseB	Rhodanase-Like Enzyme, Sulfur Transfer From Thiosulfate	1.17
sufS	Cysteine Desulfurase, Stimulated By Sufe	1.98
torD	Tora-Maturation Chaperone	1.85
tsaE	Trna(ANN) T(6)A37 Threonylcarbamoyladenosine Modification Protein	2.82
uxuA	Mannonate Hydrolase	1.93
yafF	Pseudogene	3.23
yaiX	Pseudogene	2.11
ybdD	Conserved Protein, DUF466 Family	1.64
ybjH	Predicted Protein	2.36
ydiQ	Predicted Electron Transfer Flavoprotein Subunit	2.01
yecF	Conserved Protein, DUF2594 Family	1.12
yecM	Predicted Metal-Binding Enzyme	1.36
yfdP	CPS-53 (Kple1) Prophage	2.39
yffB	Predicted Reductase, Function Unknown, Arsc Family	1.69
yfiP	Conserved Protein, DTW Domain	3.03
ygbA	Predicted Protein	1.75
yhaC	Predicted Protein	1.20
yhfZ	Conserved Protein	1.10
yiaA	Inner Membrane Protein, Yiaab Family	3.00
yigI	Conserved Protein, 4HBT Family Of Thioesterases	1.36
yjeJ	Predicted Protein	1.36
ypeC	Conserved Protein	1.86
blc	Outer Membrane Lipoprotein (Lipocalin), Cell Division And Growth Function	-1.00
copA	Copper Transporter (EC:3.6.3.4)	-2.59
cueO	Multicopper Oxidase (Laccase)	-2.03
cysK	Cysteine Synthase A, O-Acetylserine Sulfhydrolase A Subunit (EC:2.5.1.47)	-1.54
dctA	C4-Dicarboxylic Acid, Orotate And Citrate Transporter	-1.09
dppF	Dipeptide Transporter	-1.39
glcG	Conserved Protein	-2.19
hyaF	Protein Involved In Nickel Incorporation Into Hydrogenase-1 Proteins	-1.39
malF	Maltose Transporter Subunit	-1.36
phoA	Bacterial Alkaline Phosphatase	-1.00
rhsE	Pseudogene	-1.00

ssnA	Predicted Chlorohydrolase/Aminohydrolase	-2.26
tsaA	Trna-Thr(GGU) M(6)T(6)A37 Methyltransferase, SAM-Dependent	-1.88
xdhB	Xanthine Dehydrogenase, FAD-Binding Subunit	-2.23
yciW	Predicted Oxidoreductase	-2.17
yfcI	Conserved Protein	-1.57
ygbT	Multifunctional Endonuclease Cas1, CRISPR Adaptation Protein	-2.08
ygeX	2,3-Diaminopropionate Ammonia Lyase, PLP-Dependent	-1.78
ymfD	E14 Prophage	-2.42
yqeB	Conserved Protein With NAD(P)-Binding Rossman Fold	-1.94
yhiJ	Predicted Protein	-1.40

Note:

Samples were collected from three biological replicates.

p value less than 0.05 were considered significant.

Table S2 Total gene expression changes in *S. oneidensis* induced by *E. coli*

	**Extracellular electron transfer pathway – MTR pathway**	
**Gene**	**Function**	**log_2 (fold change)**
cymA	Membrane anchored tetraheme cytochrome c	0.73
mtrA	Extracellular iron oxide respiratory system periplasmic decaheme cytochrome c component	2.65
mtrC	Extracellular iron oxide respiratory system surface decaheme cytochrome c component	0.75
omcA	Extracellular iron oxide respiratory system surface decaheme cytochrome c component	1.39
mtrB	Extracellular iron oxide respiratory system outer membrane component	2.72
	**Extracellular electron transfer pathway – Flavin biosynthesis**	
**Gene**	**Function**	**log_2 (fold change)**
fccA	Periplasmic fumarate reductase	0.78
frdA	Quinol:fumarate reductase FAD-binding subunit	0.97
SO 3468	Riboflavin synthase alpha subunit RibC-like protein	4.11
ribE	Riboflavin synthase beta subunit RibE	1.26
	**Formate dehydrogenase genes**	
**Gene**	**Function**	**log_2 (fold change)**
fdhA	Formate dehydrogenase	3.03
fdhB	Formate dehydrogenase	5.81
fdhT	Formate dehydrogenase	1.23
fdhX	Formate dehydrogenase	6.75
	**Chemotaxis and motility related**	
**Gene**	**Function**	**log_2 (fold change)**
pilA	Type IV Pilin Protein PilA	2.26
pilE	Type IV Minor Pilin Protein PilE	2.14
pilY	Type IV Pili System Adhesin PilY	2.83
pilX	Type IV Minor Pilin Protein PilX	1.75
pilV	Type IV Pilin Modification Protein PilY	0.64
fliC	Cell motility, flagellar assembly proteins rod,hook and filament	1.51
motX	Flagellar rotation associated protein motx	0.41
fliD	Flagellar filament capping protein FliD	0.58
flaG	Uncharacterized flagella locus protein flag	0.11
cheA	Cell motility and bacterial chemotaxis: chemotaxis signal transduction system histidine kinase Chea	1.66
cheB	Cell motility and bacterial chemotaxis; chemotaxis signal transduction system response regulator with glutamate	3.56

	methylesterase domain CheB	
fliT	Flagella biosynthesis chaperone for flid FliT	1.65
cheZ	Chemotaxis signal transduction system chey phosphatase CheZ	0.98
cheY	Chemotaxis signal transduction system response regulator CheY	5.05
wbfD	Polysaccharide biosynthesis lipoprotein WbfD	-0.34
fliH	Flagellar assembly protein FliH	1.09
flgM	Flagellar biosynthesis anti-sigma factor FlgM	1.46
SO 1854	Outer membrane protein required for motility and nitrate resistance	-1.03
cheR	Chemotaxis signal transduction system MCP methyltransferase CheR	2.46
cheW	Chemotaxis signal transduction system adaptor protein CheW	1.58
flhF	Flagellar polar localization control system flagellar assembly determinant gtpase FlhF	0.27

Metabolism related

**Gene**	**Function**	**log_2 (fold change)**
fccA	Periplasmic Fumarate Reductase Fcca	0.78
lpxC	UDP-3-0-Acyl N-Acetylglucosamine Deacetylase Lpxc	0.51
SO 4520	Xenobiotics Biodegradation And Metabolism Like Chlorocyclohexane And Chlorobenzene Degradation And Polycyclic Aromatic Hydrocarbon Degradation	3.30
dacA	D-Alanyl-D-Alanine Carboxypeptidase Daca	0.73
pyrG	CTP Synthase Pyrg	0.51
rpoB	DNA-Directed RNA Polymerase Beta Subunit Rpob	0.33
purA	Adenylosuccinate Synthetase Pura	0.09
ppsA	Phosphoenolpyruvate Synthase Ppsa	-0.26
adhB	Guanosine Pentaphosphate Phosphatase Gppa	2.99
holC	ATP Synthase F1 Beta Subunit Atpd	1.84
gltA	Citrate Synthase Glta	-0.60
carB	Carbamoyl-Phosphate Synthase Large Subunit Carb	0.28
eno	Enolase Eno	0.71
deoD	Purine Metabolism; Purine Nucleoside Phosphorylase Deod	3.49
suhB	Inositol-Phosphate Phosphatase Suhb	0.01
sucD	Succinyl-Coa Synthase Alpha Subunit Sucd	1.29
wbpP	UDP-Glkcnac C4 Epimerase Wbpp	2.53
hemE	Uroporphyrinogen Decarboxylase Heme	1.03
atpB	ATP Synthase F0 A Subunit Atpb	-0.35
iscS	Cysteine Desulfurase Iscs	0.61
menF	Menaquinone-Specific Isochorismate Synthase Menf	2.45
guaB	Inosine-5-Monophosphate Dehydrogenase Guab	-0.29
tpiA	Triosephosphate Isomerasetpia	-0.41

aroK	Shikimate Kinase Arok	0.66
cydA	Cytochrome D Ubiquinol Oxidase Subunit I Cyda.	4.14
hemN	Coproporphyrinogen III Oxidase Oxygen-Independent Hemn	2.29
lpxD	UDP-3-O-(3-Hydroxymyristoyl) Glucosamine N-Acyltransferase Lpxd	1.02
sucC	Succinyl-Coa Synthase Beta Subunit Succ	2.07
carA	Carbamoyl-Phosphate Synthase Small Subunit Cara	-0.22
rfbB	Dtdp-Glucose-4,6-Dehydratase Rfbb	3.61
hemG	Porphyrin And Chlorophyll Metabolism; Hemg; Oxygen-Independent Protoporphyrinogen Oxidase Hemg	5.42
sdhB	Succinate Dehydrogenase Iron-Sulfur Protein Sdhb	1.09
SO 4504	Formate Dehydrogenase Associated Protein Of Unknown Function	1.28
pflA	Pyruvate Formate-Lyase 1 Activating Enzyme Pfla	0.89
aceE	Pyruvate Dehydrogenase E1 Component Acee	1.46
sdhC	Succinate Dehydrogenase Cytochrome B556 Subunit Sdhc	0.50
gabD	Succinate-Semialdehyde Dehydrogenase [NAD(P)+] Gabd	2.89
rpe	Ribulose-Phosphate 3-Epimerase Rpe	1.29

Note:

Samples were collected from three biological replicates.

p value less than 0.05 were considered significant.
